# Complementary feeding practices and associated factors among children aged 6–23 months in Pakistan

**DOI:** 10.1371/journal.pone.0247602

**Published:** 2021-02-25

**Authors:** Muhammad Ali, Muhammad Arif, Ashfaq Ahmad Shah

**Affiliations:** 1 Department of Economics, School of Social Sciences and Humanities, National University of Sciences and Technology, Islamabad, Pakistan; 2 Department of Mass Communication, School of Social Sciences and Humanities, National University of Sciences and Technology, Islamabad, Pakistan; 3 School of Management Science and Engineering, Nanjing University of Information Science and Technology (NUIST), Nanjing Jiangsu Province, P. R. China; International Centre for Integrated Mountain Development (ICIMOD), NEPAL

## Abstract

Premature mortality and undernutrition rates in Pakistan are among the highest in the world. Inadequate infant and young child feeding are the major causes of premature mortality and undernutrition. Yet, very little is known about the determinants of complementary feeding practices in Pakistan. Therefore, this study aims to identify the determinants of inadequate complementary feeding practices among children aged 6 to 23 months in Pakistan by using the latest nationally representative data from the Pakistan Demographic and Health Survey (2017–18). The results show that only 12% of children consume a minimum acceptable diet, 21% achieve minimum dietary diversity, and 38% reach minimum meal frequency. Multivariate regression analysis shows that child age, child weight at birth, mother’s access to newspapers/magazines at the individual level, wealth at the household level, and prenatal visits at the community level are significant predictors of complementary feeding practices among children aged 6–23 months in Pakistan. These findings show that, in addition to poverty alleviation, raising awareness through health practitioners, increasing access to media, and expanding access to child and maternal healthcare can improve complementary feeding practices in Pakistan. This consequently reduces premature mortality and undernutrition.

## Introduction

Undernutrition is the leading cause of under-five mortality around the world. Inadequate infant and young child feeding (IYCF) practices are the major determinants of undernutrition, optimal growth, and development, especially in the first two years of life [1]. Exclusive breastfeeding provides necessary nutrients and energy to the child, especially in the first six months of life [2]. However, as the child grows older, exclusive breastfeeding is no longer sufficient for adequate levels of energy and nutrients for the child. Therefore, the World Health Organization (WHO) recommends the introduction of appropriate complementary foods with breastfeeding after six months of birth [3]. Consumption of complementary solid, semi-solid, or soft foods with breastfeeding contributes to the healthy development of a child after the age of six months [4].

In addition, WHO recommends the adequate consumption of iron-rich food and a diversified diet with minimum recommended frequency. While complementary food replaces breastmilk, if the food is of low nutrient density, it leads to micronutrient deficiency and increased incidence of diarrhea, particularly for children between the ages of 6 and 12 months [5]. Moreover, interventions related to improvement in food intake after two years of life do not have a significant relationship with nutritional outcomes [6]. While WHO’s recommendations are applicable for all countries, they are particularly important in the case of low-income countries with high food insecurity and prevalence of malnutrition. In low-income countries, if WHO’s guidelines for minimum food requirements are followed in the first two years of a child’s life, it can reduce the death rate in children by about twenty percent [7]. Compliance with the WHO’s guidelines on a minimum acceptable diet also helps in reducing obesogenic dietary behaviors and achieving natural and healthy weight gain [8].

Globally, about one-third of the under-five deaths are attributable to undernutrition, with the majority of these deaths recorded in Asia [9]. About 21.3% of under-five children are stunted (too short for their height), 13% are underweight (too thin for their age), and 6.9% are wasted (too thin for their height) [10]. Malnutrition is responsible for sixty percent of the annual under-five deaths in developing countries [11]. About 66% of the under-five deaths are attributable to inadequate feeding practices, including undiversified and infrequent feeding [12]. South Asia bears a disproportionally high burden of malnutrition relative to other regions with the highest percentages of stunting (33.2%, about 60 million children) and wasting (14.8%, about 27 million children).

Infant and child mortality rates in Pakistan are among the highest in the world at 62 infant deaths and 74 child deaths per 1000 live births in 2018 [13]. Pakistan is also the worst performer in terms of the prevalence of malnutrition in the world. Within South Asia, the stunting and wasting rates in Pakistan (37.6% and 7.1%, respectively) are only behind the war-affected Afghanistan [10]. Rural areas bear a significantly high burden of malnutrition compared to the urban areas with 2.4 percentage points higher stunting rate, six percentage points higher underweight rate, and 3.4 percentage points higher incidence of wasting [13]. Similarly, the poor-rich divide is also quite evident; for instance, stunting, underweight, and wasting rates are significantly higher for children in the lowest wealth quintile compared to the children in the highest wealth quintile (two times, four times, and three times higher, respectively). Recent studies have shown that inadequate food consumption is one of the main determinants of stunting in Pakistan [14, 15].

Data from the Demographic and Health Survey (2017–18) shows that only 12% of under-five children in Pakistan received the recommended minimum acceptable diet in 2018, where 21% met minimum diet diversity requirement and 38% met minimum food frequency requirement [13]. Despite Pakistan’s poor performance in the sector, the literature on the determinants of child feeding practices at a national level in Pakistan is scarce and has focused on specific regions within the country (see [16] for a systematic literature review). One exception is Na et al. (2017), who used multi-level regressions to study the factors associated with complementary feeding practices in Pakistan using Demographic and Health Survey, 2012–13. However, their study is based on eight years old data, and their analyses do not differentiate between urban and rural areas. Therefore, the objectives of this study are to provide fresh evidence on the geographical distribution of child feeding practices in Pakistan at national, urban, and rural levels and to examine the relationship of child feeding practices with individual-, household-, and community-level indicators using the latest nationally representative data from Pakistan Demographic and Health Survey, 2017–18 [13]. Existing literature on the determinants of child feeding practices, including minimum dietary diversity and meal frequency, has shown that feeding practices are correlated with maternal education, maternal occupation, the gender of the child, and postnatal care [11]. Moreover, place of residence, household size, household wealth, age of the child, and place of delivery are also among the significant correlates of child feeding practices [17–19]. Recent studies have also shown that birth interval, mother’s empowerment, and exposure to media are also significant predictors of dietary diversity [20]. The variables used in this analysis are guided by the studies mentioned above.

## Methods and materials

### Sampling and data source

The 2017–18 Pakistan Demographic and Health Survey (PDHS) is implemented by the National Institute of Population Studies (NIPS) with support from the Ministry of National Health Services, Regulations, and Coordination. A nationally representative sample of 16,240 households is selected from women in the age group of 15–49 years. Stratified sampling was carried out for PDHS 2017–18 in two stages. In the first stage, Primary Sampling Units (PSUs) were selected based on probability proportional to the primary sampling unit size (580 clusters were selected). In the second stage of selection, a fixed number of 28 households was randomly drawn from every cluster by using an equal probability systematic sampling procedure. For representative data collection, all eight regions (including Punjab, Khyber Pakhtunkhwa, Balochistan, Sindh, Islamabad Capital Territory, Gilgit Baltistan, Federally Administered Tribal Areas, and Azad Jammu and Kashmir) are divided into urban and rural areas, and 16 sampling strata created. The survey of men was conducted in one-third of the sample households, and information for children aged less than five years was collected in the households selected for the men’s survey. About 15,671 households were selected for the survey, and about 11,869 households were successfully interviewed, yielding a response rate of 96%. Since complementary foods are recommended after six months of life, the analysis is restricted to children between the ages of 6 and 23 months.

### Measurement of variables

Minimum dietary diversity is based on the WHO recommendation of consuming at least four food groups out of seven to provide necessary nutrients and energy for the child to ensure normal growth. PDHS contains a module on IYCF based on the mother’s 24-hour recall of foods given to her child. The seven food groups covered in PDHS for children between 6–23 months of age include grains, roots, and tubers; legumes and nuts; dairy products (milk, yogurt); flesh foods (meat, fish, poultry and liver/organ meats; eggs; vitamin- A-rich fruits and vegetables; and other fruits and vegetables. PDHS does not ask for the quantity consumed from each food group, i.e., any quantity consumed is considered sufficient for counting.

Minimum meal frequency is defined as the proportion of children between 6–23 months of age (breastfed or otherwise) who received solid, semi-solid, or soft foods for at least the minimum number of times recommended by WHO. For non-breastfed children, milk is also considered in calculating the minimum food-frequency. The definition of “minimum” varies by age. Breastfed children between 6 and 8 months of age and between 9 and 23 months of age should consume solid or semi-solid food minimum twice a day and thrice a day, respectively. Non-breastfed children between 6 and 23 months of age should consume solid or semi-solid food at least four times a day, and also, they should intake dairy or formula milk.

A minimum acceptable diet is a combination of diet diversity and meal frequency variables where a breastfed child 6–23 months of age is considered to be receiving a minimum acceptable diet if he had at least the minimum dietary diversity and meal frequency in the last 24 hours. Similarly, a non-breastfed child 6–23 months of age is considered to be receiving a minimum acceptable diet if he has received at least two milk feedings with minimum dietary diversity (excluding milk) and minimum meal frequency in the last 24 hours.

For ease of interpretation, the explanatory variables are grouped into three categories: individual-, household-, and community-level indicators. Individual-level indicators include age (categorized as 6–11, 12–17, and 18–23 months), sex, child’s size at birth (smaller than average, average, and larger than average), receiving of vitamin A supplement, receiving of iron supplement, receiving of age-appropriate vaccination, as well as whether the subject got diarrhea recently, got fever recently, got cough recently, and birth order. Parent-related indicators include mother’s current age, mother’s age at birth, education of mother and father, mother and father’s occupation, whether the mother smokes, whether the mother has a say in her health decisions alone or jointly, exposure to media (newspaper dummy equals 1 if mother reads newspapers at least once a week, a dummy for TV equals one if mother watches television daily and a dummy for radio equals one if mother listens to radio daily), had a cesarean delivery or not, delivery at a health facility, delivery assisted by a health professional, had postnatal checkups and had at least four prenatal checkups. Household-level indicators include the type of cooking fuel (electricity, LPG, natural gas or biogas = 1), the gender of household head (male = 1), distance from the water source (within 30 minutes from dwelling = 1), wealth (poor, middle and rich), drinking water source (piped, bore, other improved, unimproved), toilet type (piped to sewer, flush to a septic tank, flush to pit latrine, other improved, unimproved, open defecation), handwashing facility with soap and water, household size, and the number of under-five children. The wealth index was generated using principal component analysis on the following indicators: accessibility to electricity; ownership of a radio, refrigerator, bicycle, motorcycle, car; no. of rooms for sleeping; floor material; roof material; and wall material. The index is divided into five quintiles and the first two quintiles are labeled “low income,” the third quintile is labeled “middle” income, and the fourth and fifth quintiles are labeled “high income.” Community-level indicators include rural-urban residence, province dummies, percentage of improved toilets, improved water, at least four prenatal visits, age-appropriate vaccination, mother empowerment, mother education, assisted delivery, cesarean delivery, postnatal visits, vitamin A supplements, and iron supplements at community (PSU) level.

### Data processing and analysis

Three complementary feeding indicators (minimum dietary diversity, minimum meal frequency, and minimum acceptable diet) were examined against a set of independent variables divided into three groups as explained above to identify the factors associated with child feeding practices in Pakistan. Statistical analyses were performed using Stata version 14.2 (Stata Corp., College Station, TX, USA). Stepwise backward selection is used to exclude the least significant variables (p>0.1) from the regressions until all the remaining variables in the final model are significant at least at 10%. Final models are further tested for multicollinearity, and variables with Variance Inflation Factor (VIF) greater than five were excluded from the final model. Logistic regressions are used to estimate multivariate relationships, and odds ratios (OR) are reported along with their 95% confidence intervals to assess the adjusted risk of independent variables.

## Results

### Sample characteristics

After data cleaning and selection of relevant age-specific cases, the weighted sample size of this analysis is 2,688 children in 453 communities. Sample characteristics by individuals, households, and community are presented in Table 1. At an individual level, about half the children were male (52%), second to fourth born (52%), and had an interpregnancy interval of 24 months or longer (49%). About 41% of the children were in the 12–17 months age group, followed by 31% in 6–11 months and 27% in 18–23 months groups. The majority of the children were currently breastfed, received age-appropriate vaccinations (65%), and had an average weight at birth (72%). Most of the mothers were between 25 and 34 years old (57%), had delivered at a health facility (71.3%), had been assisted by a skilled attendant at delivery (94%), and did not work (83%). About half of the women had primary education or higher (51%), had at least four prenatal checkups (52%), or had access to TV (47%); however, only 44% of mothers had a say in health-related decisions in their households, and 32% mothers did not have a postnatal checkup record. Most of the fathers were between 25 and 34 years of age (52%), had secondary or higher education (66%), and were involved in non-agricultural occupations (80%). At the household-level, about 85% of households had access to improved water, 79% had access to improved toilets, and 64% had handwashing facilities with soap and water. Most of the households had male heads of the household (89%) and had a water source within 30 minutes from the dwelling (92%). About 46% of the households used efficient cooking fuel (electricity, LPG, Natural gas, or biogas). At the community level, 66% of households were in rural areas, and more than half of the sample is from the Punjab province. On average, 52% of women had at least primary education (52%), had at least four prenatal visits (52%), about three-fourths of the children received vitamin A supplements (76%), and were delivered at the health facility (72%).

**Table 1:** Individual, household and community level characteristics of the study sample, Pakistan 2017–18.

	**N**	**% or mean**
**Child Characteristics**		
Male	2688	52.1
Currently Breastfed	2688	64.7
Age (months)	2688	
6–11		31.4
12–17		41.3
18–23		27.2
Birth order	2688	
Firstborn		23.7
Second to fourth		52.4
Fifth or higher		23.9
Birth interval (month)	2688	
No previous birth		23.7
<24 months		27
> = 24 months		49.3
Perceived birth weight	2675	
Smaller than average		20.9
Average		72.4
Larger than average		6.6
Received vitamin A supplementation in the past 6 months	2498	75.5
Received iron pills, sprinkles, or syrup in the last 7 days	2537	5.9
Age-appropriate vaccination	2531	64.9
Child health: had the following symptom in the past 2 weeks		
Diarrhea	2555	29.6
Fever	2556	46.6
Cough	2555	46.8
**Maternal Characteristics**		
Age (years)	2688	
15–24		28.5
25–34		57.3
35–49		14.2
Smoker	2686	5.5
Reproductive health care		
Delivered at health facility	2688	71.3
Delivery assisted by professional	2688	94.1
Cesarean delivery	2687	25.4
At least four antenatal visits	2677	52.1
Postnatal checkup of baby in 2 months	2677	32.2
Primary education or higher	2688	51.9
Occupation	2687	
Not Working		83.3
Agriculture		5.1
Non-Agriculture		11.5
Media Access: At least once a week		
Newspaper	2684	4.9
Radio	2688	4
TV	2688	47.1
Mother is involved in health-related decision making	2665	44.1
**Paternal characteristics**		
Age (years)	2663	
15–24		8.8
25–34		53.2
35–49		38
Education	2659	
No Education		29.4
At most Primary		5.1
Secondary and above		65.5
Occupation	2654	
Agriculture		17.8
Non-Agriculture		79.5
Not working		2.7
**Household characteristics**		
Male head of the household	2688	89
Household size	2688	7.8
No. of children under five	2688	2.4
Cooking fuel	2615	
Electricity, LPG, Natural Gas, Biogas		46.4
Coal, Charcoal, Wood, Straw, Crop, Dung and other		53.6
Water source	2616	
Unimproved		11.5
Piped water		25.6
Tube well/Bore		59.4
Other Improved		3.6
Water source within 30 minutes from dwelling	2592	92.3
Toilet type	2616	
Open defecation		13.3
Unimproved sanitation		7.7
Piped to sewer		26.4
Flush to septic tank		31.5
Flush to pit latrine		17.7
Other Improved		3.4
Handwashing facility with soap and water	2556	64.3
**Community Characteristics**	**PSUs**	**% or mean (SD)**
Rural Residence	453	66.6
Geographics region	453	
Punjab		52.9
Sindh		21.6
KPK		17.3
Balochistan		4.8
Islamabad		0.8
FATA		2.5
% women with atleast primary education	453	51.9 (0.4)
Mother empowerment (involved in health decisions)	453	44.2 (0.3)
Access to health care		
% children who completed age-appropriate vaccination	453	64.5 (0.3)
% delivered in health facility	453	71.3 (0.3)
% delivered with professional assistance	453	94.1 (0.1)
% caesarean delivery	453	25.4 (0.2)
% had at least 4 prenatal visits	453	52.2 (0.3)
% children had postnatal checkups	453	32.2 (0.2)
% children received Vitamin A supplements	453	75.8 (0.2)
% children received iron supplements	453	5.8 (0.1)
Water and sanitation		
% using improved toilet	453	67.2 (0.3)
% using improved water	453	87.8 (0.2)

### Complementary feeding indicators

**Fig 1:**
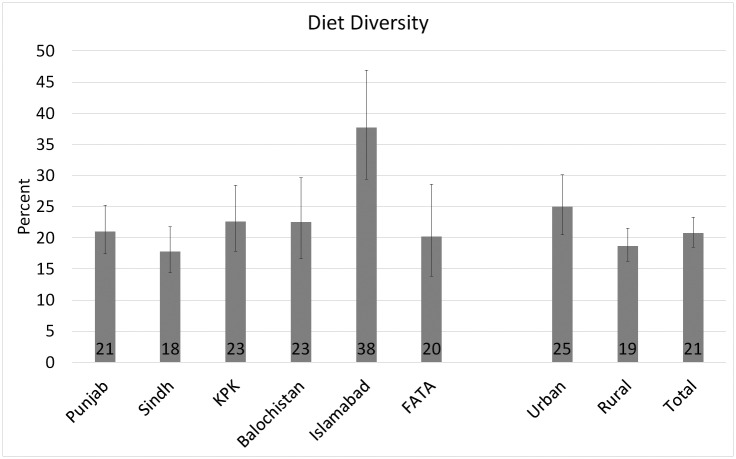
Prevalence of minimum diet diversity for children aged 6–23 months across regions and urban/rural areas in Pakistan (2017–18): 95% confidence intervals are represented by error bars. Source: Author using DHS (2017–18) data.

**Fig 2:**
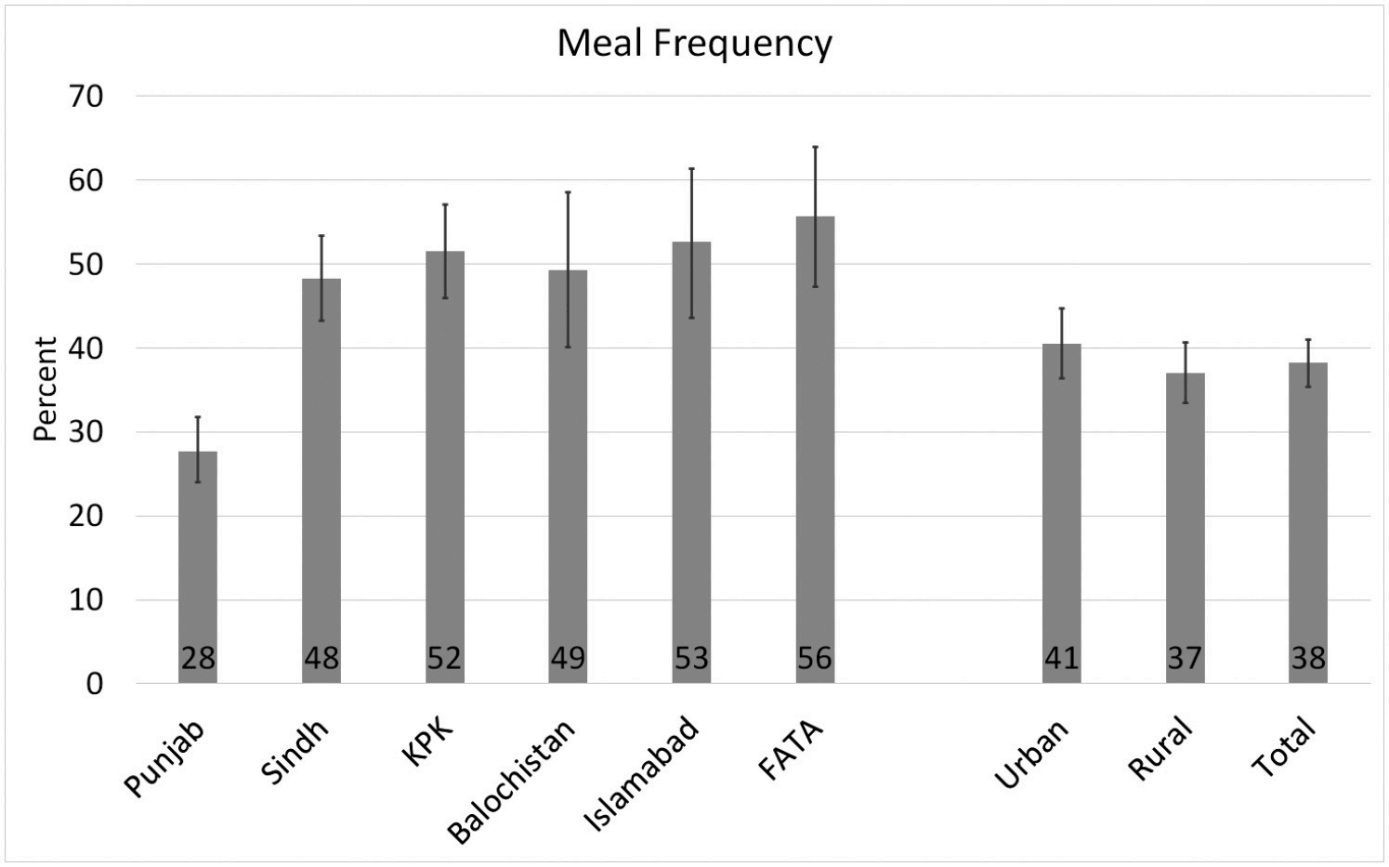
Prevalence of minimum meal frequency aged 6–23 months across regions and urban/rural areas in Pakistan (2017–18): 95% confidence intervals are represented by error bars. Source: Author using DHS (2017–18) data.

**Fig 3:**
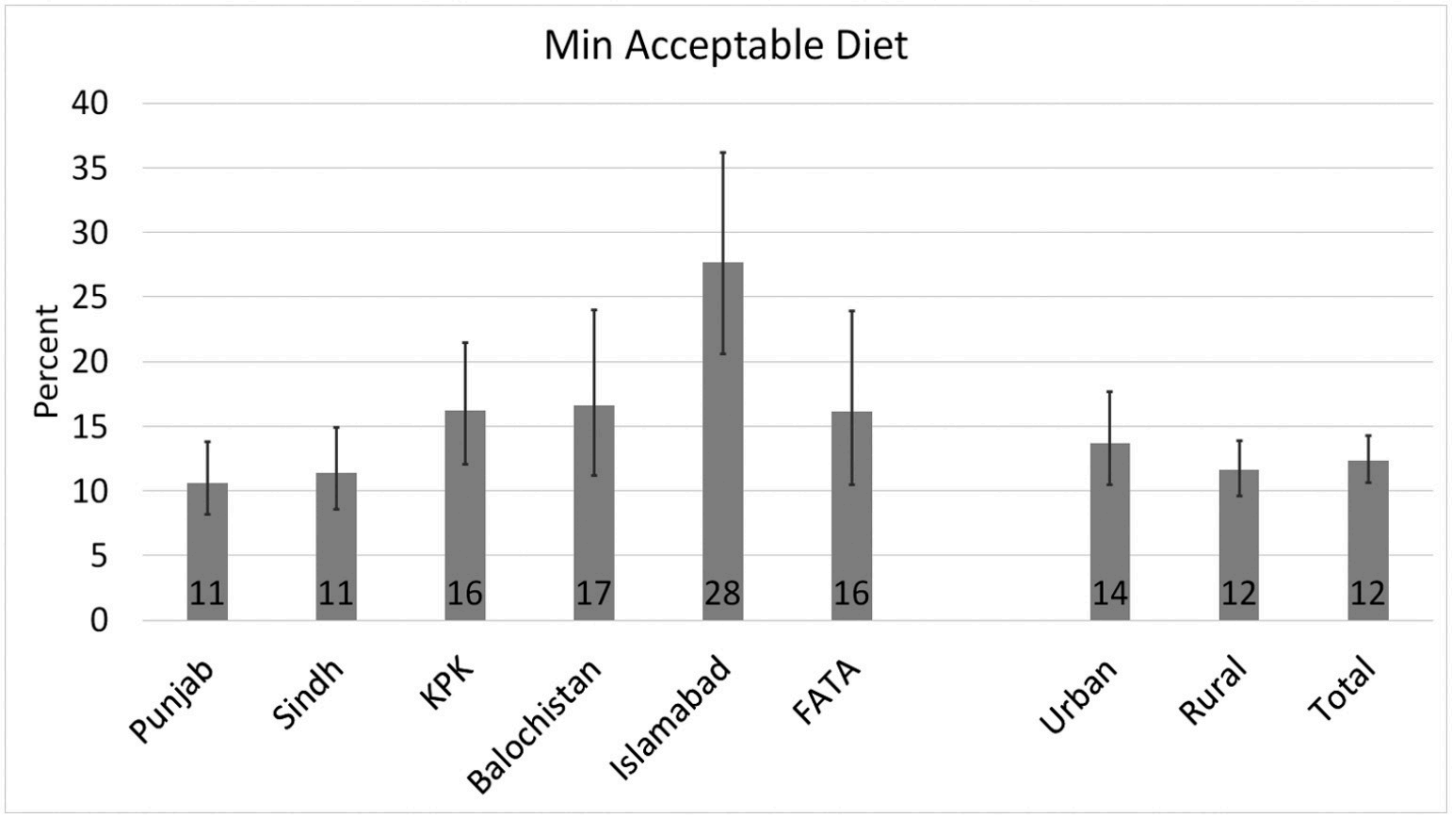
Prevalence of minimum acceptable diet aged 6–23 months across regions and urban/rural areas in Pakistan (2017–18): 95% confidence intervals are represented by error bars. Source: Author using DHS (2017–18) data.

The complementary feeding practices across provinces and urban/rural areas are presented in Figs 1 to 3, and the food group consumption by age-groups and urban/rural areas is presented in Table 2. Fig 1 shows that diet diversity score is very low in Pakistan (21%) with significant urban-rural disparity (19% in rural vs 25% in urban areas; Chi-square p-value = 0.002). Islamabad has the highest rate of minimum diet diversity (38%), and Sindh has the lowest score (18%), while the percentages of diet diversity compliance in other areas ranged between these two extremes: Balochistan (23%), Punjab (21%), KPK (23%) and FATA (20%). About 38% of the children between 6 and 23 months of age received minimum meal frequency in Pakistan (37% in rural areas and 41% in urban areas; Chi-square p-value = 0.05) (Fig 2). Compliance with the minimum meal frequency is the highest in FATA (56%) and lowest in Punjab (28%). Islamabad, KPK, Balochistan, and Sindh have minimum meal frequency compliance ranging from 48% to 53%. At 12%, the compliance with WHO’s recommendation of a minimum acceptable diet is very low in Pakistan (Fig 3) with significant variation in urban and rural areas (14% in urban and 12% in rural areas; Chi-square p-value = 0.04). About 28% of the children between 6 and 23 months of age in Islamabad received a minimum acceptable diet, followed by Balochistan (18%), KPK, and FATA (16%). Punjab and Sindh jointly house 76% of the country’s total population, and the rate of prevalence of minimum acceptable diet is the lowest in both provinces (11%), which means that majority of the children do not consume a minimum acceptable diet in Pakistan.

**Table 2:** Diet diversity, meal frequency, minimum acceptable diet and food group consumption by age and urban-rural areas.

Regions	Percentage of children in different age groups			
	6–11 Months	12–17 Months	18–23 Months	6–23 Months
Children with Diversified Diet				
Urban	34.78	19.54	22.49	25.02
Rural	35.14	9.10	13.82	18.67
Total	35.02	12.73	16.62	20.79
Children with minimum Meal Frequency				
Urban	33.74	44.18	42.34	40.47
Rural	34.36	41.84	33.05	37.03
Total	34.15	42.65	36.06	38.18
Children consuming Minimum Acceptable Diet				
Urban	18.67	12.30	10.18	13.71
Rural	21.70	6.28	7.85	11.61
Total	20.71	8.37	8.60	12.31
Children consuming Grains, Roots and tubers				
Urban	50.90	84.62	87.14	74.91
Rural	49.23	82.11	85.78	72.71
Total	49.78	82.98	86.22	73.45
Children consuming Legumes and Nuts				
Urban	3.44	10.33	9.64	8.03
Rural	4.54	9.47	8.29	7.58
Total	4.18	9.77	8.73	7.73
Children consuming Dairy				
Urban	8.49	12.21	22.16	13.69
Rural	4.72	8.16	10.13	7.62
Total	5.96	9.57	14.02	9.65
Children consuming Flesh Foods				
Urban	6.73	22.77	22.04	17.65
Rural	3.07	12.09	17.91	10.85
Total	4.27	15.81	19.25	13.13
Children consuming Eggs				
Urban	31.57	41.71	43.52	39.07
Rural	20.30	29.83	30.90	27.11
Total	24.01	33.96	34.98	31.12
Children consuming Vitamin A- rich fruits and vegetables				
Urban	8.23	22.22	23.86	18.35
Rural	8.61	20.38	27.97	18.76
Total	8.49	21.02	26.64	18.63
Children consuming Other fruits and vegetables				
Urban	15.36	38.51	42.12	32.33
Rural	14.89	30.09	33.13	26.12
Total	15.04	33.02	36.04	28.20

Source: Authors using DHS (2017–18) data.

Age-wise distribution of feeding practices (Table 2) shows that both diet diversity and minimum acceptable diet scores reduce with age, particularly in rural areas (Nonparametric trend test: z-test p-values <0.00). Compliance with minimum meal frequency does not show a linear trend (Nonparametric trend test: z-test p-values >0.1). The percentage of children satisfying minimum meal frequency shows an inverted u-shaped relationship with age as meal frequency increases when a child moves from the age group of 6–11 months to 12–17 months, but it decreases again when the child moves to 18–23 months age group. Most of the children consume starch-based diets such as grains, roots, and tubers (73%), and the percentage of children consuming starch-based diets increases with age. Eggs (31%) and Other fruits and vegetables (28%) are also among the most consumed groups with a similar relationship with age as of starch-based foods. *Legumes and nuts* group is the least consumed food group in Pakistan (8%) followed by Dairy (10%). Only about 13% of the children consume flesh foods with an increase in consumption with age except for urban areas where flesh food consumption decreases when they enter the 18–23 months age group.

#### Factors associated with minimum dietary diversity

The adjusted odds ratios from the multivariate logistic regressions for the factors associated with minimum dietary diversity compliance (95% CI) are presented (Table 3). Among individual-level characteristics, child age is a significant predictor of minimum dietary diversity. Children aged 6–11 months have two-fold, three-fold and ~4-fold higher odds of meeting minimum dietary diversity in urban areas [2.3 (1.59–3.33)], overall sample [2.96 (2.28–3.83)], and rural areas [3.97 (2.71–5.81)], respectively, as compared to the children aged 18–23 months. Children who had above average weight at birth and who took vitamin A supplements in the last six months have higher odds of meeting diet diversity, both in the full sample and rural areas. Children who received age-appropriate vaccinations have higher odds of meeting diet diversity in urban areas [1.55 (1.07–2.26)]. Among parent’s characteristics, children of mothers aged 25–34 years of age have lower odds of meeting diet diversity than those in 15–24 years and 35–49 years age groups [0.75 (0.60–0.94)], and mothers who read newspapers or magazines [1.40 (0.94–2.09)] and had cesarean delivery [1.25 (0.96–1.63)] have higher odds of complying with their child’s dietary diversity; however, above mentioned characteristics are insignificant in urban and rural regressions. Among household characteristics, lower wealth status has lower odds of compliance with dietary diversity in the full sample and rural regressions. In overall and urban regressions, children in households headed by male members are more likely to consume a diversified diet. In rural regressions, households using unimproved water source (reference: piped water) [0.53 (0.32–0.90)] and with household size between 6–9 members (reference: 1–5 members) [0.65 (0.43–0.97)] have lower odds of diet diversity compliance. Among community characteristics, a higher proportion of at least four prenatal visits at the community level is associated with higher odds of dietary diversity compliance in all three regressions, whereas postnatal checkups were only significant in urban regression [1.99 (1.09–3.62)]. The use of improved water and sanitation assisted delivery, and age-appropriate vaccinations have lower odds of meeting dietary diversity, which is against our expectations.

**Table 3:** Significant correlates (OR (95% CI)) of adequate diet diversity as per WHO recommendations using multivariate logistic regressions.

	Full Sample	P value	Rural	P value	Urban	P value
	OR (95% CI)		OR (95% CI)		OR (95% CI)	
**Child Characteristics**						
Child Age (months)						
6–11	2.96*** (2.28–3.83)	0.00	3.97*** (2.71–5.81)	0.00	2.30*** (1.59–3.33)	0.00
12–17	0.79* (0.59–1.04)	0.09	0.73 (0.47–1.11)	0.14	0.83 (0.56–1.23)	0.35
18–23	1.00		1.00		1.00	
Perceived Birth Weight						
Smaller than Average	1.00		1.00			
Average	1.13 (0.86–1.48)	0.37	1.41* (0.96–2.07)	0.08		
Larger than Average	1.85*** (1.20–2.86)	0.01	2.63*** (1.39–5.00)	0.00		
Received Vitamin A supplement—in past 6 months						
Yes	1.34** (1.06–1.69)	0.01	1.49** (1.05–2.10)	0.02		
No	1.00		1.00			
Received Age-Appropriate Vaccinations						
Yes					1.55** (1.07–2.26)	0.02
No					1.00	
**Parent Characteristics**						
Mother’s Current Age						
15–24	1.00					
25–34	0.75** (0.60–0.94)	0.01				
35–49	0.78 (0.57–1.08)	0.13				
Mother’s Occupation						
Not working					1.00	
Agriculture					11.39* (0.94–137.79)	0.06
Non Agriculture					1.20 (0.74–1.95)	0.46
Reads Newspaper or Magazine atleast once a month						
Yes	1.40* (0.94–2.09)	0.09				
No	1.00					
Delivery Mode						
Child was delivered through Cesarean						
Yes	1.25* (0.96–1.63)	0.09				
No	1.00					
Delivery Place						
Delivery in a medical/health facility						
Yes			1.63*** (1.14–2.34)	0.01		
No			1.00			
Father’s Occupation						
Not working					0.81 (0.31–2.15)	0.68
Agriculture					1.00	
Non-Agriculture					0.47** (0.24–0.91)	0.02
**Household Characteristics**						
Household Wealth Index						
Low Income	0.68** (0.49–0.94)	0.02	0.55** (0.34–0.91)	0.02		
Middle	0.75* (0.55–1.00)	0.05	0.57** (0.35–0.93)	0.02		
High Income	1.00		1.00			
Source of drinking water						
Unimproved			0.53** (0.32–0.90)	0.02		
Piped water			1.00			
Tube well/Bore			0.85 (0.58–1.25)	0.41		
Other improved			0.93 (0.52–1.67)	0.81		
Head of the household is male						
Yes	1.45* (0.98–2.14)	0.06			2.68*** (1.36–5.27)	0.00
No	1.00				1.00	
Household Size						
1–5			1.00			
6–9			0.65** (0.43–0.97)	0.04		
10+			0.85 (0.57–1.26)	0.42		
**Community Characteristics**						
% HH using Improved Water					0.37*** (0.19–0.70)	0.00
% HH using Improved Sanitation	0.54** (0.32–0.90)	0.02	0.39** (0.19–0.82)	0.01		
% Mothers with atleast 4 prenatal visits	3.55*** (2.19–5.75)	0.00	2.87*** (1.43–5.75)	0.00	4.73*** (2.45–9.16)	0.00
% Mothers who had postnatal checkups					1.99** (1.09–3.62)	0.03
% Children with age-appropriate vaccination	0.68* (0.45–1.02)	0.06			0.45** (0.23–0.88)	0.02
% Children delivered with professional assistance			0.28** (0.09–0.89)	0.03		
Region						
Punjab	1.00		1.00		1.00	
Sindh	0.94 (0.69–1.30)	0.72	0.91 (0.53–1.56)	0.73	0.83 (0.54–1.29)	0.41
KPK	1.56*** (1.15–2.11)	0.00	1.46 (0.90–2.39)	0.13	2.02*** (1.27–3.22)	0.00
Balochistan	1.48* (0.99–2.22)	0.06	2.22** (1.17–4.22)	0.01	1.27 (0.72–2.25)	0.41
Islamabad	1.67*** (1.13–2.47)	0.01	2.36** (1.15–4.82)	0.02	1.24 (0.77–2.02)	0.38
FATA	1.28 (0.83–1.97)	0.27	0.92 (0.46–1.85)	0.82	1.72 (0.79–3.73)	0.17
Constant	0.11*** (0.05–0.24)	0.00	0.40 (0.10–1.61)	0.20	0.14*** (0.04–0.46)	0.00
VIF	1.52		1.76		1.49	
N	2476		1307		1153	

* p < 0.1,

** p < 0.05,

*** p < 0.01.

#### Factors associated with minimum meal frequency

The adjusted odds of factors associated with minimum meal frequency for overall, rural and urban samples are presented in Table 4. Among individual-level characteristics, male children, children aged 12–17 months, and those with average or larger than the average size at birth have higher odds of receiving adequate meal frequency. Gender and birthweight are insignificant in urban regressions. However, children born in the order of second to fourth have 35% higher odds of meeting minimum meal frequency only in urban areas [1.35 (0.99–1.85)]. In the overall sample, none of the parents’ or household characteristics were significant. In urban and rural regressions, mothers older than 20 years are associated with higher odds of meal frequency compliance in both urban and rural regressions. In urban areas, mothers who read newspapers or magazines have 70% higher odds of meal frequency compliance [1.70 (1.07–2.70)] whereas those who listen to the radio and those who had assisted delivery had lower odds of achieving minimum meal frequency. Among household characteristics in urban areas, the use of efficient fuel is associated with higher odds of meeting minimum meal frequency [1.41 (0.99–2.01)]. Among community characteristics, postnatal checkups and age-appropriate vaccinations at the community level have higher odds of a child receiving minimum meal frequency in overall and urban regressions. In rural regressions, at least four prenatal visits have three-fold higher odds of meal frequency compliance [3.32 (2.02–5.46)], while communities with a higher percentage of assisted deliveries and empowered mothers have lower odds of satisfying the minimum meal frequency requirement. In urban regressions, the percentage of cesarean deliveries have lower odds [0.43 (0.22–0.84)], whereas the percentage of children who received vitamin A supplements have higher odds of meeting minimum meal frequency [1.90 (1.06–3.39)].

**Table 4:** Significant correlates (OR (95% CI)) of adequate meal frequency as per WHO recommendations using multivariate logistic regressions.

	Full Sample	P value	Rural	P value	Urban	P value
	OR (95% CI)		OR (95% CI)		OR (95% CI)	
**Child Characteristics**						
Child Sex						
Male	1.17* (0.99–1.37)	0.07	1.29** (1.03–1.63)	0.03		
Female	1.00					
Child Age (months)						
6–11	0.90 (0.72–1.11)	0.32	0.96 (0.71–1.29)	0.79		
12–17	1.27** (1.03–1.56)	0.02	1.54*** (1.16–2.06)	0.00		
18–23	1.00		1.00			
Birth Order						
Firth born					1.00	
Second to fourth					1.35* (0.99–1.85)	0.06
Fifth or higher					1.33 (0.89–1.99)	0.17
Perceived Birth Weight						
Smaller than Average	1.00		1.00			
Average	1.51*** (1.22–1.87)	0.00	1.49*** (1.11–1.99)	0.01	1.77*** (1.24–2.55)	0.00
Larger than Average	1.91*** (1.32–2.77)	0.00	1.89** (1.12–3.18)	0.02	2.49*** (1.40–4.45)	0.00
**Parent Characteristics**						
Mother’s Age at Birth						
Less than 20			0.75** (0.59–0.96)	0.02	1.25 (0.95–1.65)	0.11
20–29						
More than 30			1.03 (0.49–2.18)	0.94	2.43** (1.13–5.24)	0.02
Reads Newspaper or Magazine atleast once a month						
Yes					1.70** (1.07–2.70)	0.02
No					1.00	
Listens to Radio						
Yes					0.56* (0.30–1.05)	0.07
No					1.00	
Child delivery was assisted by the trained professional						
Yes					0.53* (0.25–1.12)	0.09
No					1.00	
Father’s Education						
No Education					1.00	
Atmost Primary					0.34*** (0.16–0.70)	0.00
Secondary and above					0.77 (0.55–1.09)	0.14
**Household Characteristics**						
Cooking Fuel						
Electricity, LPG, Natural Gas, Biogas					1.41* (0.99–2.01)	0.06
Coal, Charcoal, Wood, Straw, Crop, Dung					1.00	
**Community Characteristics**						
% Cesarean deliveries					0.43** (0.22–0.84)	0.01
% Children Received Vitamin Supplements					1.90** (1.06–3.39)	0.03
% Mothers with atleast 4 prenatal visits			3.32*** (2.02–5.46)	0.00		
% Mothers who had postnatal checkups	2.01*** (1.48–2.72)	0.00			2.11** (1.13–3.93)	0.02
% Children with age-appropriate vaccination	1.71*** (1.20–2.44)	0.00			1.64* (0.93–2.87)	0.09
% Children delivered with professional assistance			0.41** (0.17–0.99)	0.05		
% Mothers who are empowered			0.46** (0.24–0.87)	0.02		
Region						
Punjab	1.00		1.00		1.00	
Sindh	2.62*** (2.04–3.36)	0.00	3.86*** (2.69–5.55)	0.00	2.02*** (1.35–3.02)	0.00
KPK	3.51*** (2.74–4.48)	0.00	2.53*** (1.68–3.82)	0.00	2.70*** (1.74–4.21)	0.00
Balochistan	3.31*** (2.41–4.55)	0.00	3.94*** (2.46–6.31)	0.00	2.69*** (1.60–4.54)	0.00
Islamabad	2.88*** (2.04–4.06)	0.00	2.26*** (1.23–4.13)	0.01	2.79*** (1.73–4.52)	0.00
FATA	4.66*** (3.38–6.42)	0.00	3.77*** (2.21–6.44)	0.00	2.89*** (1.47–5.69)	0.00
Constant	0.10*** (0.07–0.16)	0.00	0.35** (0.14–0.91)	0.03	0.12*** (0.04–0.38)	0.00
VIF	1.36		1.57		1.46	
N	2512		1349		1,085	

* p < 0.1,

** p < 0.05,

*** p < 0.01.

#### Factors associated with minimum acceptable diet

The adjusted odds ratios from the multivariate logistic regressions for the factors associated with the minimum acceptable diet (95% CI) are presented in (Table 5). Among individual-level characteristics, children between 6–11 months of age, who are born in fifth or higher-order, who were larger than average in size at birth, and those who received vitamin A supplements in the past six months have higher odds of receiving minimum acceptable diet, whereas those who had a fever in past two weeks have lower odds of receiving minimum acceptable diet. Birth order is insignificant in rural regressions, and vitamin A supplementation is insignificant in urban regressions. Among parent characteristics, mothers younger than 25 years, who work in the non-agricultural sector, who read newspapers or magazines, and who had cesarean delivery have higher odds of meeting minimum acceptable diet requirements. The mother’s occupation and cesarean delivery are insignificant in both urban and rural regressions, whereas the mother’s age and mother’s access to newspapers or magazines are insignificant in rural regressions. Children of fathers who have at least primary education have twice the odds of receiving a minimum acceptable diet [2.48 (1.23–4.98)]. Among household characteristics, children who live in households that use efficient fuel for cooking (full sample), improved water (full sample), or have a male head of the household (full and urban sample) have higher odds of meeting minimum acceptable diet. In rural regressions, household size between 6–9 members is associated with lower odds [0.56 (0.35–0.89)], while in urban regressions, households with ten or more members are associated with higher odds of meeting minimum acceptable diet. Among community characteristics, utilizing improved water (full and urban sample) and improved sanitation (full and rural sample) have lower odds of meeting minimum acceptable diet. In communities with a higher percentage of women receiving at least four prenatal checkups, have at least ~4-folds higher odds of consuming a minimum acceptable diet in all three samples.

**Table 5:** Significant correlates (OR (95% CI)) of minimum acceptable diet as per WHO recommendations using multivariate logistic regressions.

	Full Sample	P value	Rural	P value	Urban	P value
	OR (95% CI)		OR (95% CI)		OR (95% CI)	
**Child Characteristics**						
Child Age (months)						
6–11	3.43*** (2.47–4.77)	0.00	4.17*** (2.62–6.65)	0.00	2.66*** (1.69–4.20)	0.00
12–17	1.12 (0.79–1.60)	0.53	1.07 (0.64–1.79)	0.80	1.12 (0.69–1.81)	0.65
18–23	1.00		1.00			
Birth Order						
Firth born	1.00				1.00	
Second to fourth	1.02 (0.74–1.40)	0.92			0.99 (0.65–1.52)	0.98
Fifth or higher	1.68** (1.09–2.60)	0.02			1.92** (1.07–3.46)	0.03
Perceived Birth Weight						
Smaller than Average	1.00		1.00		1.00	
Average	1.48** (1.05–2.09)	0.02	1.66** (1.04–2.65)	0.03	1.22 (0.75–1.99)	0.42
Larger than Average	2.53*** (1.51–4.23)	0.00	2.68*** (1.27–5.64)	0.01	2.08** (1.02–4.26)	0.04
Received Vitamin A supplement—in past 6 months						
Yes	1.52*** (1.14–2.01)	0.00	1.75*** (1.16–2.63)	0.01		
No	1.00		1.00			
Had fever in past two weeks						
Yes	0.68*** (0.53–0.88)	0.00	0.73* (0.51–1.04)	0.08	0.63*** (0.45–0.89)	0.01
No	1.00		1.00		1.00	
**Parent Characteristics**						
Mother’s Current Age						
15–24	1.00				1.00	
25–34	0.68** (0.50–0.92)	0.01			0.58** (0.39–0.88)	0.01
35–49	0.54*** (0.34–0.85)	0.01			0.55* (0.29–1.04)	0.06
Mother’s Occupation						
Not working	1.00					
Agriculture	0.60 (0.21–1.73)	0.35				
Non Agriculture	1.80*** (1.21–2.67)	0.00				
Reads Newspaper or Magazine atleast once a month						
Yes	1.93*** (1.23–3.02)	0.00			2.11*** (1.26–3.54)	0.00
No	1.00				1.00	
Delivery Mode						
Child was delivered through Cesarean						
Yes	1.48** (1.08–2.03)	0.02				
No	1.00					
Father’s Education						
No Education			1.00			
Atmost Primary			2.48** (1.23–4.98)	0.01		
Secondary and above			1.31 (0.88–1.96)	0.18		
**Household Characteristics**						
Cooking Fuel						
Electricity, LPG, Natural Gas, Biogas	1.32* (0.96–1.82)	0.09				
Coal, Charcoal, Wood, Straw, Crop, Dung 1.00						
Source of drinking water						
Improved	1.62** (1.04–2.52)	0.03				
Unimproved	1.00					
Head of the household is male						
Yes	1.56* (0.94–2.58)	0.09			3.44** (1.32–8.96)	0.01
No	1.00				1.00	
Household Size						
1–5			1.00		1.00	
6–9			0.56** (0.35–0.89)	0.01	1.39 (0.83–2.35)	0.21
10+			0.75 (0.48–1.18)	0.22	1.74** (1.04–2.91)	0.04
**Community Characteristics**						
% HH using Improved Water	0.33*** (0.18–0.59)	0.00			0.43** (0.20–0.92)	0.03
% HH using Improved Sanitation	0.47** (0.25–0.90)	0.02	0.38** (0.17–0.86)	0.02		
% Mothers with atleast 4 prenatal visits	4.40*** (2.53–7.64)	0.00	4.69*** (2.17–10.16)	0.00	5.43*** (2.60–11.34)	0.00
Region						
Punjab	1.00		1.00		1.00	
Sindh	1.19 (0.80–1.77)	0.39	1.25 (0.68–2.30)	0.47	0.93 (0.54–1.61)	0.79
KPK	2.60*** (1.77–3.82)	0.00	2.34*** (1.42–3.87)	0.00	2.73*** (1.57–4.73)	0.00
Balochistan	2.68*** (1.64–4.37)	0.00	3.17*** (1.59–6.32)	0.00	1.69 (0.86–3.32)	0.12
Islamabad	2.97*** (1.88–4.68)	0.00	2.86*** (1.29–6.35)	0.01	2.36*** (1.35–4.12)	0.00
FATA	2.18*** (1.30–3.67)	0.00	1.61 (0.85–3.03)	0.14	2.50** (1.06–5.87)	0.04
Constant	0.02*** (0.01–0.06)	0.00	0.02*** (0.01–0.06)	0.00	0.01*** (0.00–0.05)	0.00
VIF	1.52		1.45		1.48	
N	2375		1290		1,155	

* p < 0.1,

** p < 0.05,

*** p < 0.01.

## Discussion

In this study, we examined the factors associated with minimum dietary diversity, minimum meal frequency, and minimum acceptable diet among children between 6 and 23 months of age in Pakistan by using the most recent nationally representative data from Demographic and Health Survey, 2017–18. About a quarter of the children met diet diversity criteria, two-fifth met minimum meal frequency criteria, and, marginally, more than one in ten children met the minimum acceptable diet criteria. The percentage of children consuming a minimum acceptable diet in Pakistan is ranked among the lowest in the South Asian region; only marginally better than India (Sri Lanka 62% [21], Nepal 36% [22], Bangladesh 23% [23], Afghanistan 16% [24], and India 9.6% [25]). Poor dietary intake among children in Pakistan is, therefore, a serious concern as consumption of a diversified diet is associated with adequate micronutrient intake and a lower risk of stunted growth among children in developing countries [26–28].

All food groups, except Legumes and Nuts, show an increase in consumption with age, which contradicts the hypothesis of substitution among food types across age groups in the recent study on Pakistan (Na et al., 2017 [29]). Most children consume a starch-based diet (grains, roots, and tubers = 73%) in Pakistan. Legumes and Nuts, and Dairy products are among the least consumed food groups. Among non-breastfed children, 12% of children consume dairy products in Pakistan, which is ranked lowest in the South Asian region (Sri Lanka 52.6% [21], Afghanistan 45.1% [24], India 26.2% [25], Nepal 16% [22], and Bangladesh 12.6% [23]). The least consumed food groups in Pakistan are essential for fatty acids, micronutrients, bioactive compounds, foods sourced from animals for improved digestibility, which are necessary for optimal child development [30, 31]. Low dairy consumption among non-breastfed children in Pakistan is surprising because Pakistan is the 4^th^ largest milk producer in the world, and milk is the main livestock commodity, the growth of which has been increasing [32]. Moreover, the consumption of dairy products is not correlated with livestock ownership. The possible reasons could be that the poor livestock owners use milk for their livelihood instead of feeding their children. It is also possible that mothers are not aware of the importance of feeding dairy products to their children.

The results of multivariate regression analysis show that children in the age group of 6–11 months have higher odds of achieving minimum diet diversity and minimum acceptable diet than children in the older age groups. Our findings are in line with previous studies in Bangladesh [33], Sri Lanka [34], and Pakistan [29]. The adjusted odds associated with child-age are higher in rural areas. The possible explanation could be that mothers are particularly careful about their child’s feeding requirements in the first year whereas, in later years, they rely on the child’s demands about the type of food and frequency of food rather than following the recommended guidelines, especially in rural areas where literacy and awareness levels are usually low. Perceived child weight at birth is also associated with adequate child feeding practices; children who were of average or above-average size at birth have higher odds of receiving minimum diet diversity, meal frequency, and acceptable diet. Child weight is not significantly correlated with minimum dietary diversity in urban areas suggesting that diet patterns do not vary by the weight of a child at birth in urban areas. It could be because of the belief that a child with a smaller than the average size at birth may not digest several types of foods [29].

Mother’s age is also a significant predictor of minimum dietary diversity and minimum acceptable diet except in rural areas. Adjusted odds ratios show that children of older mothers have lower odds of receiving recommended diet, and it may be explained by the willingness of young mothers to comply with the recommendations of modern research, and they may not subscribe to the erroneous cultural beliefs as strongly as the older mothers do. Also, reading a newspaper or a magazine is positively correlated with minimum dietary diversity and acceptable diet in the full sample and urban sample, whereas it is only significant for minimum meal frequency in the urban sample. The insignificance of newspaper access in rural areas may be because the percentage of women who read newspapers or magazines in urban areas is about thrice as high as those in rural areas (9% vs. 3%: Chi-square p-value = 0.00). Nevertheless, increasing access to the newspaper can improve complementary feeding in Pakistan.

Contrary to the findings of previous studies on developing countries, a mother’s education is not significantly associated with complementary feeding practices [11, 16, 33, 34]. Similar findings were reported by Na et al. (2017) [29], who used a similar multi-level model used in this study and explained that differences in the results could be arising because of a higher number of explanatory variables used in the analysis. The positive correlation of a mother’s education with child feeding practices is generally interpreted as a mother’s level of awareness in the literature [34]. Since access to the newspaper is a significant predictor of child feeding practices in this analysis, it could also be the reason why a mother’s education is not a significant predictor of child feeding practices.

Among household-level characteristics, the wealth of the household is a significant predictor of minimum dietary diversity, and gender of the household head is a significant predictor of dietary diversity and minimum acceptable diet among children between 6–23 months of age, except in rural areas. Previous studies on Pakistan have shown that poverty is a significant determinant of child feeding practices [16, 29, 35]. The results indicate that a household’s affordability to purchase necessary food is one of the prerequisites to achieve dietary diversity [33]. Studies on complementary feeding practices in Bangladesh have shown that dietary diversity is generally lower in food insecure households [36]. Both wealth and gender of the household head are insignificant predictors of minimum meal frequency in all three samples, probably because meeting meal frequency is less resource-dependent. Previous studies have also shown that wealth predicts minimum diet diversity and acceptable diet but not minimum meal frequency [29, 33, 34].

At the community level, child feeding practices varied significantly by region. Among other characteristics, the number of prenatal visits at the community level was positively associated with complementary feeding practices, which may be explained by the messages delivered to the mothers by the health care providers during those visits or by the community members even if the mother did not visit the health care provider herself. Counseling, media campaigns, and well-organized state health services related to IYCF, such as training and messages delivered during prenatal and postnatal checkups, have shown to improve the minimum acceptable diet in Bangladesh [37] and Sri Lanka [34]. Such awareness-raising and behavior change campaigns can also improve feeding practices in Pakistan.

## Limitation of the study

The following limitations of the study should be considered when interpreting the results. This study is based on cross-sectional data; therefore, the causal inference may not be strong. Moreover, the DHS survey collects IYCF data on a single 24-h recall basis, which can be affected by respondent vias in which there is a tendency to give socially desirable answers. The survey also does not provide information on the quantity and quality of food consumed, due to which nutritional adequacy of the food consumed cannot be measured.

## Conclusions and recommendations

Overall, the current study shows that only 12% of the children in the age group of 6–23 months receive the minimum acceptable diet which is significantly lower than other countries in the region such as India, Bangladesh, and Sri Lanka. Poor complementary food practices are widespread across regions. Our results show several factors associated with complementary feeding practices, including a child’s age, weight at birth, the wealth of the household, and prenatal visits at the community level. Some of the findings differ for rural and urban areas suggesting that the policy measures should be context-specific. Targeting the poor households and those with poor access to prenatal care is recommended to improve complementary feeding practices in Pakistan.
